# High-resolution crystal structure of a polyextreme GH43 glycosidase from *Halothermothrix orenii* with α-l-arabinofuranosidase activity

**DOI:** 10.1107/S2053230X15003337

**Published:** 2015-02-19

**Authors:** Noor Hassan, Lokesh D. Kori, Rosaria Gandini, Bharat K. C. Patel, Christina Divne, Tien Chye Tan

**Affiliations:** a School of Biotechnology, KTH Royal Institute of Technology, Stockholm, Sweden; b Department of Medical Biochemistry and Biophysics, Karolinska Institutet, Stockholm, Sweden; c Microbial Gene Research and Resources Facility, School of Natural Sciences, Griffith University, Brisbane, QLD 4111, Australia; d Department of Biochemistry and Molecular Biology, Baylor College of Medicine, Houston, TX 77030, USA

**Keywords:** glycoside hydrolase, five-bladed β-propeller, arabinofuranosidase, *Halothermothrix orenii*, halothermophile

## Abstract

A gene from the heterotrophic, halothermophilic marine bacterium *Halothermothrix orenii* has been cloned and overexpressed in *Escherichia coli*. This gene encodes the only glycoside hydrolase of family 43 (GH43) produced by *H. orenii*. The crystal structure of the *H. orenii* glycosidase was determined by molecular replacement and refined at 1.10 Å resolution. As for other GH43 members, the enzyme folds as a five-bladed β-propeller. The structure features a metal-binding site on the propeller axis, near the active site. Based on thermal denaturation data, the *H. orenii* glycosidase depends on divalent cations in combination with high salt for optimal thermal stability against unfolding. A maximum melting temperature of 76°C was observed in the presence of 4 *M* NaCl and Mn^2+^ at pH 6.5. The gene encoding the *H. orenii* GH43 enzyme has previously been annotated as a putative α-l-arabinofuranosidase. Activity was detected with *p*-nitrophenyl-α-l-arabinofuranoside as a substrate, and therefore the name *Ho*Araf43 was suggested for the enzyme. In agreement with the conditions for optimal thermal stability against unfolding, the highest arabinofuranosidase activity was obtained in the presence of 4 *M* NaCl and Mn^2+^ at pH 6.5, giving a specific activity of 20–36 µmol min^−1^ mg^−1^. The active site is structurally distinct from those of other GH43 members, including arabinanases, arabinofuranosidases and xylanases. This probably reflects the special requirements for degrading the unique biomass available in highly saline aqueous ecosystems, such as halophilic algae and halophytes. The amino-acid distribution of *Ho*Araf43 has similarities to those of mesophiles, thermophiles and halophiles, but also has unique features, for example more hydrophobic amino acids on the surface and fewer buried charged residues.

## Abstract 2

The crystal structure of the *H. orenii* glycosidase was determined by molecular replacement and refined at 1.10 Å resolution.

## 1. Introduction   

Carbohydrate-based polymers offer a large renewable natural resource, and extensive efforts have been invested in investigating how biomass may be used to harness energy through its conversion to fermentable sugars by environmentally sustainable enzymatic processes. The most prominent decomposers of lignocellulosic biomass are bacteria and fungi, which typically produce intricate and complex networks of hydrolytic (glycoside hydrolases; GHs) and oxidative enzymes for the complete solubilization of cellulose, hemicelluloses and lignins. In this context, the genomes of thermophilic anaerobic microbes present a treasure trove of genetic information, including a large repertoire of genes encoding intracellular and extracellular glycoside hydrolases, some of which could possess useful biochemical properties suitable for exploitation in improved or new enzymatic bioprocesses.

The marine bacterium *Halothermothrix orenii* of the order Haloanaerobiales is interesting as an obligate halothermophile, and is also the first truly thermophilic halophile to be described whose complete genome has been sequenced (Mijts & Patel, 2001 ▶; Mavromatis *et al.*, 2009 ▶). This strictly anaerobic bacterium was first isolated from the 40–60 cm bottom sediment layer of the warm saline lake Chott El Guettar in Tunisia (Cayol *et al.*, 1994 ▶). This habitat supports the optimal growth of *H. orenii* at a temperature of 60°C (range 45–68°C) with 1.7 *M* NaCl (range 0.7–3.4 *M*) and within the pH range 5.5–8.2, with optimal growth between pH 6.5 and 7.0 (Cayol *et al.*, 1994 ▶).

The *H. orenii* genome contains only a single gene encoding a family 43 glycoside hydrolase (CAZy GH43; http://www.cazy.org; Lombard *et al.*, 2014 ▶), which contrasts with, for instance, the genome of the soil saprophyte *Cellvibrio japonicus*, which contains 14 GH43 genes (DeBoy *et al.*, 2008 ▶). The GH43 family mainly includes hemicellulases, and the large number of GH43 genes in *C. japonicus* may reflect the need for a wide variety of hemicellulases for the efficient degradation of complex plant cell-wall material. Since information is lacking regarding substrate availability in the various sediment layers of Chott El Guettar, we may only speculate that this saline environment offers biomass of a less variable nature, probably mostly of algal origin, which could explain the lack of catalytic diversification of GH43 enzymes in *H. orenii*. To date, the crystal structures of 28 unique GH43 members have been determined, mainly including bacterial β-xylosidases, xylanases, α-l-arabinofuranosidases and arabinanases (http://www.cazy.org). Based on sequence similarity to other GH43 arabinofuranosidases (EC 3.2.1.55), the gene encoding the *H. orenii* glycosidase (Gene ID 7314382; UniProt accession No. B8CZV1) was suggested to be a putative α-l-arabinofuranosidase (Kori, 2012 ▶). Here, we report the cloning, overexpression, high-resolution crystal structure determination and preliminary biochemical characterization of the *H. orenii* GH43 glycosidase.

## 2. Experimental procedures   

### 2.1. Cloning, expression and purification   

The gene encoding the *H. orenii* glycosidase was cloned into the pNIC28-Bsa4 vector with a cleavable His_6_ tag and a *Tobacco etch virus* (TEV) protease cleavage site at the N-terminus (Savitsky *et al.*, 2010 ▶) using ligation-independent cloning (Doyle, 2005 ▶). The recombinant plasmid was transformed into *Escherichia coli* strain BL21(DE3) and the protein was overexpressed for 16–18 h at 18°C following induction with 0.2 m*M* isopropyl β-d-1-thiogalactopyranoside. The cells were lysed in 20 m*M* HEPES pH 7.0, 150 m*M* NaCl (lysis buffer) using an Avestin Emulsiflex-C3 system. The gene product was purified from the lysate using Ni–NTA agarose resin (Invitrogen) eluted with lysis buffer containing 350 m*M* imidazole. The His_6_ tag and imidazole were removed by dialysis of the sample overnight in a dialysis bag (12–14 kDa molecular-weight cutoff) containing TEV protease and 20 m*M* HEPES pH 7.0, 150 m*M* NaCl. The sample was rerun through the Ni–NTA resin and the flowthrough containing the target protein was collected and concentrated. To remove remaining Ni^2+^ ions, 20 m*M* EDTA and 20 m*M* EGTA were added and a final step of gel filtration was performed using a HiLoad 26/60 Superdex 200 prep-grade column (GE Healthcare Life Sciences) equilibrated with 20 m*M* HEPES pH 7.0, 150 m*M* NaCl. Eluted fractions containing the *H. orenii* glycosidase were collected in a 96-well deep-well microplate, pooled and concentrated using a Vivaspin centrifugal concentrator (10 kDa molecular-weight cutoff).

### 2.2. Stability assay using Thermofluor screening   

The effects of various parameters on the thermal stability against unfolding of the GH43 glycosidase were investigated using Thermofluor screening (Ericsson *et al.*, 2006 ▶). Specifically, the influence of pH (5.5–9.0), metal ions (2 m*M* Mn^2+^, Mg^2+^, Ca^2+^ and Na^+^) and salt (0, 0.1, 0.5, 1.0, 2.0, 4.0 and 5.0 *M* NaCl) on the melting temperature was assessed. Reaction mixtures consisted of 1 µl 23 mg ml^−1^ protein in 10 m*M* bis-tris buffer pH 5.5, 1 µl SYPRO Orange dye 25× stock solution (diluted 1:40 with water) and 23 µl screen solution to give a total volume of 25 µl reaction mixture per well. The reaction mixtures were added to a 96-well thin-wallled PCR plate (Bio-Rad) and the plate was sealed with Optical Quality Sealing Tape (Bio-Rad) and heated in an iCycler iQ Real Time PCR Detection System (Bio-Rad) from 20 to 90°C in steps of 1°C, during which changes in fluorescence were monitored with a charge-coupled device (CCD) camera. The wavelengths for excitation and emission were 490 and 575 nm, respectively.

### 2.3. Screening of aryl-glycoside substrates and thin-layer chromatography   

Enzyme activity was initially screened in a high-throughput format at 50°C in 50 m*M* bis-tris buffer pH 6.5 using a 96-well deep-well plate format with the following compounds (all from Sigma–Aldrich): *p*-nitrophenyl-α-d-glucopyranoside (*p*NP-αGlc), *p*-nitrophenyl-β-d-glucopyranoside (*p*NP-βGlc), *o*-nitrophenyl-β-d-glucopyranoside (*o*NP-βGlc), *p*-nitrophenyl-α-d-galactopyranoside (*p*NP-αGal), *p*-nitrophenyl-β-d-galactopyranoside (*p*NP-βGal), *o*-nitrophenyl-β-d-galactopyranoside (*o*NP-βGal), *p*-nitrophenyl-α-l-arabinofuranoside (*p*NP-αAraf), *p*-nitrophenyl-α-l-arabinopyranoside (*p*NP-αArap), *p*-nitrophenyl-β-l-arabinopyranoside (*p*NP-βArap), *p*-nitrophenyl-α-d-xylopyranoside (*p*NP-αXyl) and *p*-nitrophenyl-β-d-xylopyranoside (*p*NP-βXyl). Activity was tested in the absence and presence of 2 m*M* metal chloride salts (Ca^2+^, Mg^2+^ and Mn^2+^) and at different NaCl concentrations (0, 1, 3 and 4 *M* NaCl).

The enzyme reactions were performed in volumes of 100 µl, including 90 µl buffer solution (50 m*M* bis-tris pH 6.5) with the appropriate concentrations of salt and metal and 8 µl 19 m*M* substrate. The buffer mixtures were placed at 50°C for 30 min to equilibrate before the enzyme was added. The reactions were started by adding 2 µl 12 mg ml^−1^ enzyme. After 2, 4 and 24 h, 100 µl sodium carbonate was added to inactivate the enzyme and to stabilize the chromophore in its anionic 4-nitrophenolate form. Samples of 150 µl were withdrawn and the absorbance was measured at 420 nm (*o*NP) or 405 nm (*p*NP). Calibration curves were prepared for *o*NP and *p*NP as standards at two selected NaCl concentrations (0 and 4 *M*) using the aforementioned reaction conditions but without adding enzyme. The *p*NP concentration range used was 0–80 m*M*. For *o*NP, a molar extinction coefficient of 4600 *M* ^−1^ cm^−1^ was used to calculate the amount of generated *o*NP.

For a repeated activity assay, the same protocol was used as above but only for those conditions that showed the highest enzyme activity in the initial screen. This time, both EDTA/EGTA-treated and untreated enzyme were used. To address the problems associated with inflated absorbance values owing to oxidation and secondary reactions of Mn^2+^ under alkaline conditions, the absorbance values for the reactions were taken before and after adding sodium carbonate. Graphs were prepared using *GraphPad Prism*.

For thin-layer chromatography (TLC) analysis, 2 µl 12 mg ml^−1^ *H. orenii* glycosidase was mixed with 8 µl 10 m*M* substrate (α-l-arabinofuranopentaose or lactose) in 50 m*M* bis-tris pH 6.5 to a final volume of 100 µl and incubated at 40°C for 4 h. The composition of the reactions (substrates and products) was analyzed using TLC. The samples from the reaction with lactose as the substrate were run on Silica gel 60 plates (Merck Millipore), while for the α-l-arabinofurano­pentaose samples HPTLC plates with Silica gel 60 matrix (Merck Millipore) were used. A mixture of *n*-butanol:isopropanol:ethanol:water (2:3:3:2) was used as the mobile phase. The plates were developed with a thymol solution (0.5 g thymol dissolved in 95 ml ethanol and 5 ml sulfuric acid) and heated at 120°C for 15 min.

### 2.4. Crystallization, data collection and structure determination   

Crystallization conditions for the *H. orenii* glycosidase were screened in 96-well Corning 3550 plates using the sitting-drop vapour-diffusion method and Mosquito Crystal robotics (TTP Labtech). Initial hits were optimized using 24-well Intelli-Plate 24 plates (Art Robbins Instrument). Well diffracting crystals grew at 298 K from 1 µl protein solution (178 mg ml^−1^ in 20 m*M* HEPES pH 7.0, 150 m*M* NaCl) mixed with 1 µl reservoir solution consisting of 0.1 *M* HEPES pH 6.2, 0.16 *M* potassium thiocyanate, 25%(*w*/*v*) polyethylene glycol 3350. The crystals belonged to space group *P*2_1_, with unit-cell parameters *a* = 44.13, *b* = 73.88, *c* = 87.52 Å, β = 94.26° and two molecules per asymmetric unit.

Prior to data acquisition, the crystal was flash-cooled in liquid nitrogen. Diffraction intensities to 1.10 Å resolution were collected on the microfocus beamline I24 at Diamond Light Source, UK equipped with a Dectris PILATUS 6M pixel detector and Oxford Danfysik/SESO bimorph-type mirrors. The data were indexed, processed and scaled using the *XDS* package (Kabsch, 2010 ▶). An initial *Phyre*2 analysis (Kelley & Sternberg, 2009 ▶) returned endo-1,4-β-xylanase from *Bacteroides thetaiotaomicron* VPI-5482 (PDB entry 3kst; Joint Center for Structural Genomics, unpublished work) as the best candidate to use as a search probe for molecular-replacement calculations. Phasing was performed by molecular replacement using *AutoMR* as implemented in the *PHENIX* suite (Adams *et al.*, 2010 ▶) with one monomer of the 3kst model. A clear solution was obtained and model building was performed using *Coot* (Emsley & Cowtan, 2004 ▶) guided by 2*F*_o_ − *F*_c_ electron-density maps. *PHENIX* was used for refinement against the maximum-likelihood target, including *xyz* refinement, real-space refinement, riding H atoms and refinement of individual anisotropic temperature factors. Data-collection and refinement statistics are given in Table 1 ▶. All figures showing structural details were generated with *PyMOL* (DeLano, 2002 ▶). Coordinates and structure factors have been deposited in the PDB as entry 4qqs.

### 2.5. Analysis of amino-acid composition   

To determine the distribution of each amino acid (on the surface or buried), the surface accessibility in Å^3^ was calculated using *DSSP* (Kabsch & Sander, 1983 ▶). The relative surface accessibility (RelAcc) was calculated by comparing the surface accessibility of each amino acid with the highest value observed. According to the definition of Fukuchi *et al.* (2003 ▶), an amino acid was considered to be surface-exposed if it showed a RelAcc value of greater than 25%. The percentage of each amino-acid type in the interior or on the surface was calculated with respect to the total number of interior or surface-exposed amino acids. To allow a direct comparison with the results of Fukuchi and coworkers, the amino acids were classified as apolar (Val, Ile, Leu, Met, Phe, Trp and Tyr), polar (Asn, Gln, Ser and Thr), basic (Lys and Arg), acidic (Asp and Glu) or other (Ala, Cys, Gly, Pro and His).

## 3. Results and discussion   

### 3.1. Overall structure and the active site   

The final model was refined with riding H atoms at 1.10 Å resolution with *R* and *R*_free_ values of 0.122 and 0.145, respectively. The deposited model includes riding H atoms, and the asymmetric unit contains two protein molecules (molecule *A*, residues 4–315; molecule *B*, residues 3–315), three HEPES molecules (one associated with molecule *A* and two with molecule *B*), one sodium ion per protein molecule and 1032 water molecules.

Based on primary and tertiary structure, the *H. orenii* glycosidase is a member of family 43 of inverting glycoside hydrolases, with accession No. cl14647 and sequence cluster cd08772 (superfamily GH43_62_32_68) in the Conserved Domains Database (CDD). Specifically, the *H. orenii* GH43 glycosidase belongs to subfamily cd08991 (GH43_bXyl_2) of the GH43 family. The members of this subfamily mainly include uncharacterized GH43 glycosidases and putative β-xylosidases.

**Figure 1:**
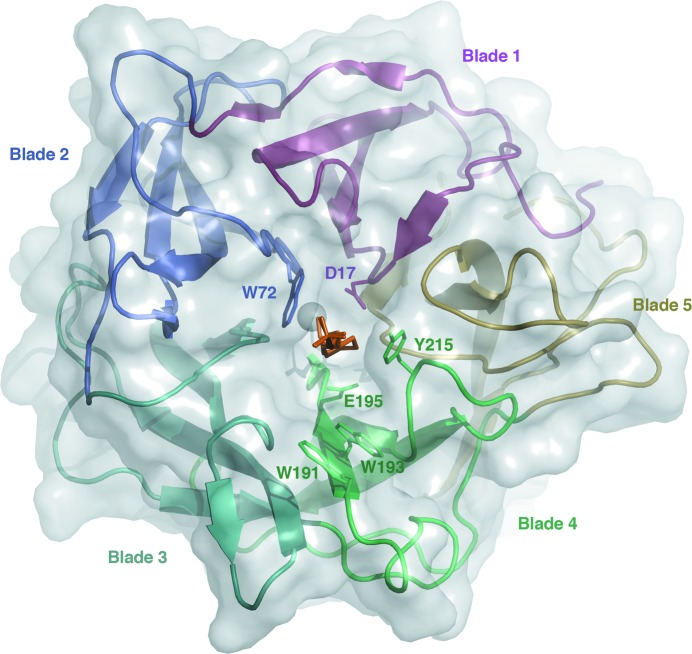
Ribbon representation showing the five-bladed β-propeller of *Ho*Araf43 with the bound HEPES molecule and the sodium ion at the central axis of the propeller.

**Figure 2:**
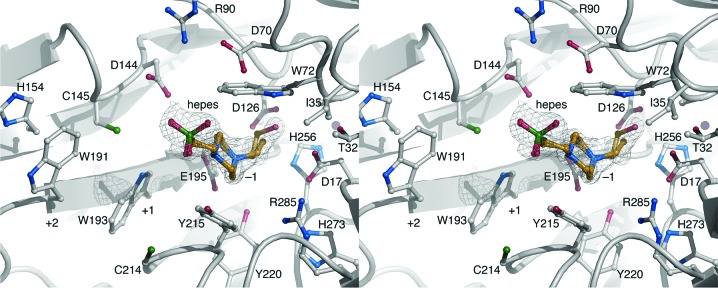
Wall-eyed stereo image showing the binding of a disordered HEPES molecule in subsite −1 superimposed on the σ_A_-weighted 2|*F*_o_| − |*F*_c_| map contoured at 0.7σ.

The enzyme folds as a five-bladed β-propeller similar to those described for inverting GH43 enzymes of clan GH-F (Fig. 1 ▶). Each blade is composed of a four-stranded antiparallel β-sheet repeated five times from the N-terminus to the C-terminus: blade 1, residues 14–59; blade 2, residues 69–120; blade 3, residues 125–179; blade 4, residues 190–244; blade 5, residues 255–306. As inferred from homology to related GH43 enzymes, the catalytic −1 subsite is situated at the bottom of a pocket formed at the ‘top’ of the β-propeller (Fig. 1 ▶). The conserved catalytic nucleophile is placed on blade 4 (Glu195), the catalytic base (Asp17) on blade 1 and Asp126 on blade 3 (Fig. 2 ▶). In homologous GH43 enzymes, the carboxylate group of Asp126, which is positioned between the nucleophile and the base, has been suggested to help modulate the p*K*_a_ of the nucleophile and participate in substrate binding (Nurizzo *et al.*, 2002 ▶). On one side of the −1 subsite, Trp72 (blade 2) forms a conserved sugar-binding platform. In the present structure, the piperazine ring of the HEPES buffer molecule is wedged between Trp72 and Tyr215 in subsite −1, but does not form a planar stacking interaction with the aromatic rings (Fig. 2 ▶). Moreover, Arg285 and Tyr215 should be able to contribute to binding in the −1 subsite. Additional subsites are present but display fundamental differences to other GH43 enzymes of known structure (see §[Sec sec3.5]3.5 below). Importantly, Trp193 and Trp191 (blade 4) may be able to offer interactions with a polysaccharide backbone or branching sugar units in subsites +1 and +2, respectively (Fig. 2 ▶). Besides the two tryptophan residues, Cys214 (+1), Asp189 (+2) and Ser217 (+2) may contribute to substrate binding.

### 3.2. Metal-binding preference   

**Figure 3:**
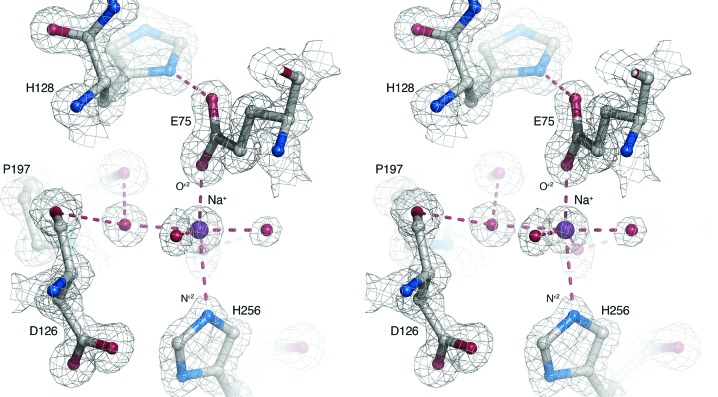
Wall-eyed stereo image showing the metal-binding site with a modelled sodium ion (purple) octahedrally coordinated by Glu75, His256 and four water molecules. The σ_A_-weighted 2|*F*_o_| − |*F*_c_| map contoured at 2.0σ is shown.

A metal-binding site is situated at the central axis of the propeller, immediately below the −1 subsite. Two types of cation, Na^+^ and K^+^, were present during crystallization of the *H. orenii* glycosidase, which are also the relevant cytoplasmic cations responsible for counterbalancing the high osmotic pressure exerted by the naturally saline medium. The metal ion present in the structure is represented by a 20σ peak in the σ_A_-weighted difference Fourier map with coordination to five ligands (Fig. 3 ▶): Glu75 O^∊2^ (2.1 Å) and four water O atoms. The imidazole group of the nearby His256 (blade 5) is positioned as a possible sixth ligand, but is 2.8 Å from the metal ion. The coordination distances for Na^+^–carboxylate O and Na^+^–imidazole N (target distances 2.42 and 2.38 Å, respectively; Harding, 2002 ▶) are considerably shorter than those for K^+^–carboxylate O and K^+^–imidazole N (target distances 2.84 and 2.80 Å, respectively; Harding, 2002 ▶). Additionally, Na^+^ and K^+^ prefer different coordination numbers, *i.e.* six and eight, respectively. Thus, based on the composition of the crystallization medium, the observed coordination number and the metal–ligand coordination distances for the bound metal, we modelled the metal ion as Na^+^. It should be emphasized that K^+^ is still possible. Neither of these metals may, however, represent the biologically relevant metal for the enzyme. We also investigated the effect of various cations on the relative thermal stability against unfolding by means of Thermofluor screening (Ericsson *et al.*, 2006 ▶). The results show that the enzyme is indeed stabilized in the presence of divalent cations (Supplementary Fig. S1). At pH 7.0, adding 2 m*M* Mn^2+^ increases the melting temperature (*T*_m_) of the enzyme by 5.5°C from 63 to 68.5°C (Supplementary Fig. S1*a*).

The metal-binding site in the *H. orenii* GH43 glycosidase is strategically positioned at the propeller axis to provide stabilizing interactions between blade 2 (Glu75) and blade 5 (His256) across the axis, which probably explains the increase in melting temperature in the presence of divalent cations. As discussed above, the metal ion bound in the crystal structure is most likely to be Na^+^ or K^+^, but the binding site would be able to favourably accommodate any of the stabilizing metal ions, *i.e.* Mn^2+^, Mg^2+^ or Ca^2+^. The metal observed in the structure is six-coordinated with one elongated interaction between the metal and His256 N^∊2^ (Fig. 3 ▶), which suggests that the physiologically and functionally relevant metal may be any of Mn^2+^, Mg^2+^ or Ca^2+^. Coordination number six is common for chelated Mn^2+^, Mg^2+^ and Ca^2+^ (Harding, 2001 ▶). Ca^2+^ tends to prefer a longer coordination distance to imidazole N atoms than Mn^2+^ or Mg^2+^, and imidazole nitrogen ligands are generally rare for Mn^2+^ (Harding, 2001 ▶). Additionally, the Glu75 O^∊2^ atom coordinates the metal monodentately (specifically anti-monodentate coordination), which is especially common for Mg^2+^, but is also common for protein ligands that coordinate Mn^2+^ and Ca^2+^ (Harding, 2001 ▶).

Some GH43 members have been confirmed to depend on divalent cations for activity. For instance, the two-domain endo-acting arabinanase from *Thermotoga petrophila* (*Tp*ABN; PDB entries 4kc7 and 4kc8; Santos *et al.*, 2014 ▶) is Ca^2+^-dependent, but with only backbone carbonyl groups and water molecules as metal ligands. Of the available GH43 structures, only the *B. thetaiotaomicron* endo-1,4-β-xylanase with bound Na^+^ (PDB entry 3kst; Joint Center for Structural Genomics, unpublished work), the exo-α-1,5-l-arabinofuranosidase *Sa*Araf43A with bound Ca^2+^ (PDB entry 3akh; Fujimoto *et al.*, 2010 ▶) and the *Clostridium acetobutylicum* β-xylosidase Abf2 with bound Mg^2+^ (PDB entry 3k1u; Midwest Center for Structural Genomics, unpublished work) show the same coordination geometry as the *H. orenii* GH43 enzyme with Glu O^∊2^ and His N^∊2^ as metal ligands. However, the dependence of these enzymes on various metal ions for structural stability or catalytic activity has not been reported.

Our results show that divalent cations improve the structural stability of the *H. orenii* glycosidase, especially in the presence of high salt, which agrees well with the hot, saline conditions under which this enzyme operates. Taken together, the structural observations regarding the metal-binding site suggest that it is well designed for binding any of the three stabilizing divalent metal ions Mn^2+^, Mg^2+^ or Ca^2+^.

### 3.3. Salt tolerance   

Since the *H. orenii* glycosidase is produced by a halophile, it is expected that the thermal stability against unfolding will depend on the salt concentration (Supplementary Fig. S1*b*). A pronounced Δ*T*_m_ value of 10°C, from 63 to 73°C, was observed at 4.0 *M* NaCl and pH 6.0–6.5. The *T*_m_ value is increased further by the cumulative stabilizing effects of divalent cations and NaCl (Supplementary Fig. S1*c*). At pH 6.0–6.5, the *T*_m_ value increases to 75–76°C in the presence of 2 m*M* MnCl_2_ with 4.0 *M* NaCl. For CaCl_2_ the most stabilizing condition is observed at pH 7.0 and 4.0 *M* NaCl, resulting in a *T*_m_ value of 74.5°C. In agreement with the optimal pH for the growth of *H. orenii*, the enzyme displays optimal stability between pH 6.5 and 7.0, which is the pH range where Mn^2+^ provides the highest degree of structural stabilization. The *H. orenii* GH43 glycosidase is an intracellular enzyme (no export signal is present), and bacteria of the order Halo­anarobiales are known to manage osmotic stress using the ‘salt-in’ strategy, where the molar concentrations of K^+^ and Na^+^ are maintained in the cytoplasm to match those in the surrounding medium (Oren, 2006 ▶). Thus, the increased thermal stability to unfolding at higher salt concentration agrees well with the strategy employed by *H. orenii*.

### 3.4. Substrate screening   

The initial activity screen using various *o*- and *p*-nitrophenyl glycosides did not provide definitive answers regarding substrate specificity. Low activity was observed after an extended incubation time (24 h) with the substrates *p*NP-αAraf, *p*NP-αArap, *p*NP-βArap, *p*NP-βGal, *p*NPαGlu, *p*NPβGlu and *o*NPβGlu (Supplementary Fig. S2). These substrates were selected for a second activity screen using the conditions that showed the highest activity for each substrate: *p*NP-*α*Araf (4 *M* NaCl, Mn^2+^; 4 *M* NaCl, Mg^2+^), *p*NP-αArap (3 *M* NaCl, Mg^2+^; 0 *M* NaCl, Mn^2+^), *p*NP-*β*Arap (0 *M* NaCl, Mg^2+^), *p*NP-βGal (1 *M* NaCl, Mg^2+^), *p*NP-αGlu (1 *M* NaCl, no metal; 0 *M* NaCl, Mn^2+^), *p*NP-βGlu (0 *M* NaCl, Mn^2+^) and *o*NP-βGlu (0 *M* NaCl, Mn^2+^). Both EDTA/EGTA-treated and untreated enzyme were used. The results from the repeated experiment confirmed and strengthened the assumption that *p*NP-αAraf is a substrate of the *H. orenii* GH43 glycosidase (Supplementary Fig. S3).

The highest activity was obtained for *p*NP-αArap in the presence of 4 *M* NaCl and Mn^2+^, and activity was lost in the absence of metal. For this condition, a slightly higher activity was observed for enzyme that had been pretreated with EDTA and EGTA compared with untreated enzyme. By taking absorbance readings before and after the addition of sodium carbonate, lower and upper estimates of the specific activity for the arabinofuranosidase activity were obtained (Table 2 ▶). The specific activity is within the range ∼7–17 µmol min^−1^ mg^−1^ for 4 *M* NaCl/Mg^2+^ and within the range ∼20–36 µmol min^−1^ mg^−1^ for 4 *M* NaCl/Mn^2+^. These values can be compared with a commercial source of α-l-arabino­furanosidase (Megazyme), which has a specific activity of ∼32 µmol min^−1^ mg^−1^ for *p*NP-αAraf. TLC analysis of reactions run during 3 h incubation of the enzyme with either α-l-arabinofuranopentaose or lactose as a substrate did not reveal any hydrolysis products.

### 3.5. Structural comparison to other GH43 enzymes   

Structure-based similarity analyses were performed using the refined model of the *H. orenii* glycosidase and the *DaliLite* (http://ekhidna.biocenter.helsinki.fi/dali_server; Holm & Rosenström, 2010 ▶) and *PDBeFold* (http://www.ebi.ac.uk/msd-srv/ssm; Krissinel & Henrick, 2004 ▶) servers. In agreement with the initial *Phyre*2 analysis, both *PDBeFold* and *DaliLite* returned PDB entry 3kst (Joint Center for Structural Genomics, unpublished work) as the best structural match.

To further analyze the possible function and substrate specificity, available crystal structures of GH43 enzymes were examined. Although the overall fold is indeed similar, the precise details of the substrate-binding region differ significantly between the enzymes. These differences are exemplified in Supplementary Fig. S4, where the *H. orenii* glycosidase structure has been superimposed with the closest structural homologue *B. thetaiotaomicron* endo-1,4-β-xylanase (PDB entry 3kst; Joint Center for Structural Genomics, unpublished work), as well as with representative GH43 enzymes in complex with carbohydrate ligands: *C. japonicus* exo-α-1,5-l-arabinanase *Cj*Arb43A in complex with arabinohexaose (PDB entry 1gye; Nurizzo *et al.*, 2002 ▶), *S. avermitilis* exo-α-1,5-l-arabinofuranosidase *Sa*Araf43A in complex with α-l-arabinofuranotriose (PDB entry 3akh; Fujimoto *et al.*, 2010 ▶), *Bacillus subtilis* arabinoxylan α-1,3-l-arabinofuranohydrolase in complex with xylotriose (PDB entry 3c7f; Vandermarliere *et al.*, 2009 ▶), *B. subtilis* endo-α-1,5-l-arabinanase Abn2 in complex with arabinotriose (PDB entry 2x8s; de Sanctis *et al.*, 2010 ▶), *C. japonicus* α-1,2-l-arabinofuranosidase Abf43A in complex with α-1,3-arabinofuranose-substituted α-1,5-l-arabinotriose (PDB entry 3qef; Cartmell *et al.*, 2011 ▶), *Humicola insolens* *Hi*AXHd3 in complex with xylotriose (PDB entry 3zxk; McKee *et al.*, 2012 ▶) and *Geobacillus stearo­thermophilus* β-xylosidase XynB3 in complex with xylobiose (PDB entry 2exj; Brüx *et al.*, 2006 ▶).

Only four residues appear to be well conserved among the 48 GH43 structures examined (*H. orenii* GH43 sequence numbers): Glu195 (the catalytic nucleophile), Asp17 (the catalytic base), Trp72 and Asp126 (Fig. 2 ▶). Beyond these side chains, larger variations occur both in the sequence and in the conformations of the loops forming the substrate-binding region (Supplementary Fig. S4). The lack of structural consensus prevents the assignment of substrate specificity to the *H. orenii* GH43 glycosidase based on homologous structures; however, a slightly higher level of active-site structure similarity exists for the *B. thetaiotaomicron* endo-1,4-β-xylanase (PDB entry 3kst; Joint Center for Structural Genomics, unpublished work), the exo-α-1,5-l-arabinofuranosidase *Sa*Araf43A (PDB entry 3akh; Fujimoto *et al.*, 2010 ▶) and the *C. acetobutylicum* β-xylosidase Abf2 (PDB entry 3k1u; Midwest Center for Structural Genomics, unpublished work). To conclude, we find that the only GH43 glycosidase produced by *H. orenii* shares a common catalytic mechanism with other known GH43 members, but that the substrate-binding region is distinct. The enzyme displays activity with *p*NP-αAraf as a substrate, but no detectable activity on *p*NP-αXyl or *p*NP-βXyl, which suggests that it is an α-1,5-l-arabinofuranosidase and not an α- or β-xylosidase. We therefore propose that the *H. orenii* GH43 glycosidase is referred to as *Ho*Araf43. However, the lack of degradation products from α-l-arabinofuranopentaose and the low sequence and structural similarity of the substrate-binding region in *Ho*Araf43 to other known GH43 enzymes, including arabinofuranosidases, indicate that although the natural substrate for *Ho*Araf43 is likely to contain arabinofuranose units, the natural substrate is probably more complex.

### 3.6. Amino-acid composition analysis   

The interior and exterior amino-acid distributions have been analyzed for proteins produced by bacteria and archaea that thrive in different environments (Fukuchi & Nishikawa, 2001 ▶; Fukuchi *et al.*, 2003 ▶). The polyextremophilic *Ho*Araf43 shares features with mesophiles, thermophiles and halophiles (Table 3 ▶): (i) the percentage of hydrophobic core amino acids is similar to that of halophiles, (ii) the percentage of polar amino acids on the surface correlates with that of the average mesophilic/nonhalophilic protein and (iii) the percentages of surface-exposed positively and negatively charged residues are identical to those of thermophilic proteins. While these similarities are noted, there are some differences (Table 3 ▶): (i) the percentage of hydrophobic residues on the surface is higher in *Ho*Araf43, (ii) the percentage of buried polar residues is higher, (iii) the percentage of buried basic residues is lower and (iv) the distribution of other amino acids (Ala, Cys, Gly, Pro and His) in the interior is similar to halophiles, although slightly higher in *Ho*Araf43.

## 4. Conclusions   

Thermostable and robust glycoside hydrolases are highly relevant to a number of biotechnological applications, including lignocellulose biomass processing and bioconversion to produce value-added sugars from inexpensive bulk resources. In this work, we have shown that the only GH43 enzyme produced by *H. orenii* is unique within this family in that (i) it is polyextreme with an unusual distribution of amino acids, (ii) it displays pronounced stabilization in the presence of high salt and divalent cations, (iii) the active site shows unique details that are distinct from other hemicellulases of known structure in this enzyme family and (iv) the enzyme is able to hydrolyze *p*NP-αAraf. The weak but distinct activity with *p*NP-αAraf justifies the classification of this *H. orenii* glycosidase as an α-1,5-l-arabinofuranosidase and its naming *Ho*Araf43. Information about the composition of the bottom sediment of Chott El Guettar is lacking, which complicates the search for the true substrate of the enzyme. However, further and wider screening of substrates may offer additional clues to the role of this intriguing enzyme and provide information regarding the potential usefulness of this enzyme in biotechnological applications.

## Supplementary Material

PDB reference: *Ho*Araf43, 4qqs

Supporting Information.. DOI: 10.1107/S2053230X15003337/tt5064sup1.pdf

## Figures and Tables

**Table 1:** Data and refinement statistics for *Ho*Araf43 Values in parentheses are for the outer shell.

Data statistics	
Space group	*P*2_1_
Molecules per asymmetric unit	2
Wavelength ()	0.9795
Unit-cell parameters	
*a* ()	44.13
*b* ()	73.88
*c* ()	87.52
()	90
()	94.26
()	90
Resolution range ()	44.011.10 (1.201.10)
*R*_merge_ (%)	2.9 (22.4)
*R*_meas_ (%)	3.2 (24.6)
No. of measured reflections	1404460 (273638)
No. of unique reflections	213964 (46299)
Mean *I*/(*I*)	30.2 (7.7)
Completeness (%)	94.5 (89.3)
Multiplicity	6.6 (5.9)
CC_1/2_ † (%)	100 (96.9)
Wilson *B* factor (^2^)	10.0
Crystallographic refinement	
*R* factor/*R*_free_ (%)	12.2/14.5
Total No. of non-H atoms	6121
No. of protein atoms	5026
Other molecules/ions	3 HEPES, 2 Na
No. of waters	1032
R.m.s. deviation from standard geometry	
Bond lengths ()	0.009
Bond angles ()	1.33
Average *B* factors (^2^)	
Protein	12.0
Water	26.0
Ramachandran statistics	
Allowed region (%)	96.2
Favoured region (%)	99.8
Outliers (%)	0.2

† CC_1/2_ is the percentage correlation between intensities from random half data sets. The values given represent correlations significant at the 0.1% level (Karplus Diederichs, 2012 ▶). Shells with CC_1/2_ exceeding 50% have been included.

**Table 2:** Specific activity of *Ho*Araf43 on *p*NP-Araf

	Specific activity† (molmin^1^mg^1^)	
Reaction conditions	Before adding Na_2_CO_3_	After adding Na_2_CO_3_
4*M* NaCl, 2m*M* Mg^2+^, EDTA/EGTA-treated *Ho*Araf43	6.68 0.32	17.38 0.20
4*M* NaCl, 2m*M* Mg^2+^, untreated *Ho*Araf43	6.69 1.2	15.86 0.56
4*M* NaCl, 2m*M* Mn^2+^, EDTA/EGTA-treated *Ho*Araf43	20.32 0.49	36.20 5.6
4*M* NaCl, 2m*M* Mn^2+^, untreated *Ho*Araf43	20.78 1.7	22.89 7.0

† Expressed as the arithmetic mean and the standard error of the mean.

**Table 3:** Comparison of amino-acid composition

	Mesophiles and nonhalophiles†		Thermophiles†		Halophiles†		Halothermophile (*H. orenii* GH43 glycosidase‡)	
Amino-acid class§ (%)	Interior	Surface	Interior	Surface	Interior	Surface	Interior	Surface
Apolar	45.6	18.1	46.9	20.2	41.5	15.1	39.2	**25.0**
Polar	15.3	21.0	13.4	15.6	14.9	17.5	**19.8**	21.0
Basic	6.0	16.7	7.1	20.1	5.8	10.9	**3.3**	20.0
Acidic	7.2	21.0	7.8	23.0	8.6	28.6	6.6	23.0
Other	25.9	3.1	24.8	21.1	29.1	28.0	31.1	**11.0**

† Values from Fukuchi *et al.* (2003 ▶).

‡ Values that are different in the *H. orenii* GH43 enzyme with respect to the other groups are highlighted in bold.

§ Classification: apolar, Val, Ile, Leu, Met, Phe, Trp, Tyr; polar, Asn, Gln, Ser, Thr; basic, Lys, Arg; acidic, Asp, Glu; other, Ala, Cys, Gly, Pro, His.
