# Supplementary material for: High-resolution crystal structure of a polyextreme GH43 glycosidase from Halothermothrix orenii with α-l-arabinofuranosidase activity
Source: Acta Crystallogr F Struct Biol Commun. 2015 Feb 19;71(Pt 3):338–45. doi: 10.1107/S2053230X15003337 (PMC4356313; doi:10.1107/S2053230X15003337)
Supplement: Supplementary file 1 [file f-71-00338-sup1.pdf]

Acta Cryst F

STRUCTURAL BIOLOGY COMMUNICATIONS

Volume 71 (2015)

Supporting information for article:

High-resolution crystal structure of a polyextreme GH43 glycosidase from *Halothermothrix orenii* with α-l-arabinofuranosidase activity

Noor Hassan, Lokesh D. Kori, Rosaria Gandini, Bharat K. C. Patel, Christina Divne and Tien Chye Tan

Supplmentary information

High-resolution crystal structure of a polyextreme GH43 glycosidase from *Halothermothrix orenii* with α-L-arabinofuranosidase activity

Noor Hassan,[a,b] Lokesh D. Kori,[c,d] Rosaria Gandini,[a,b] Bharat K. C. Patel,[c] Christina Divne,[a,b] and Tien Chye Tan[a,b*]

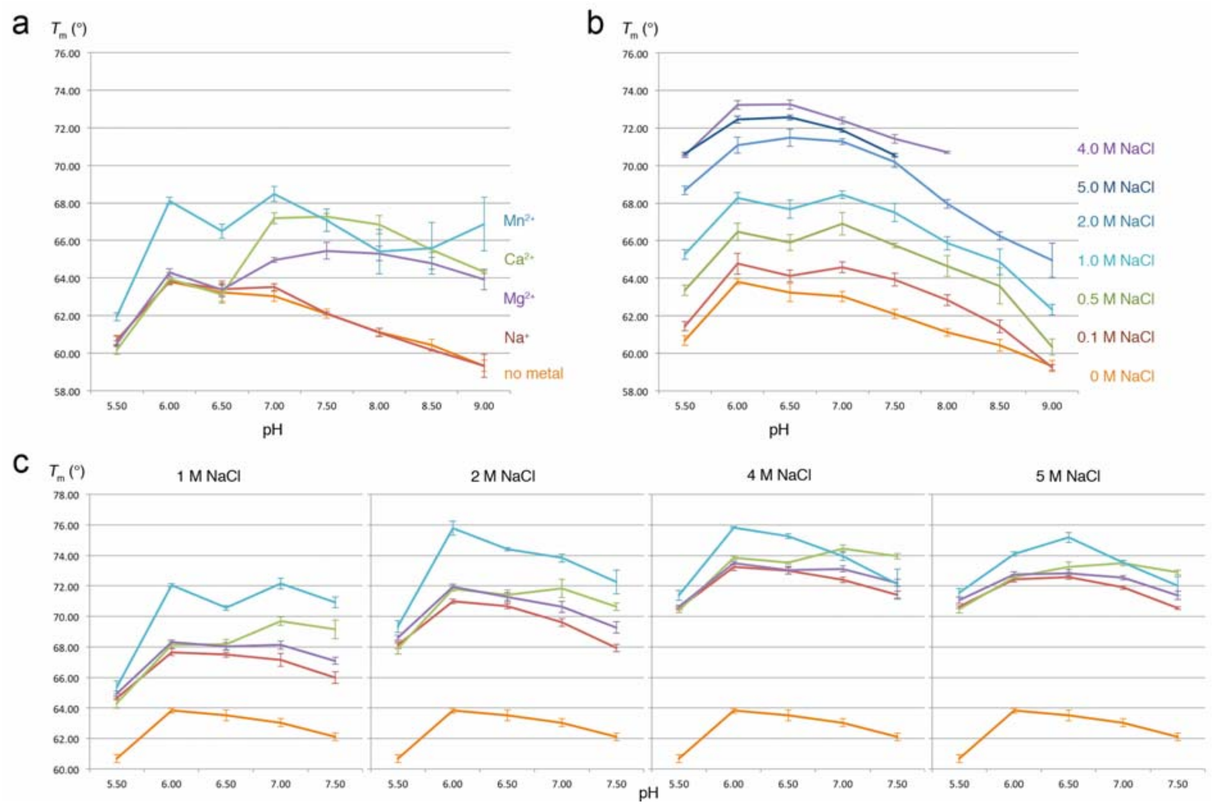

Supplementary Figure S1. Melting temperature of unfolding (T_m) as a function of (*a*) 2 mM metal ion (Mn^{2+}, Ca^{2+}, Mg^{2+} and Na^+), (*b*) NaCl concentration, and (*c*) the combined effect of NaCl concentration and metal ion (orange, no metal; red, Na^+; purple, Mg^{2+}; green, Ca^{2+}; blue, Mn^{2+}).

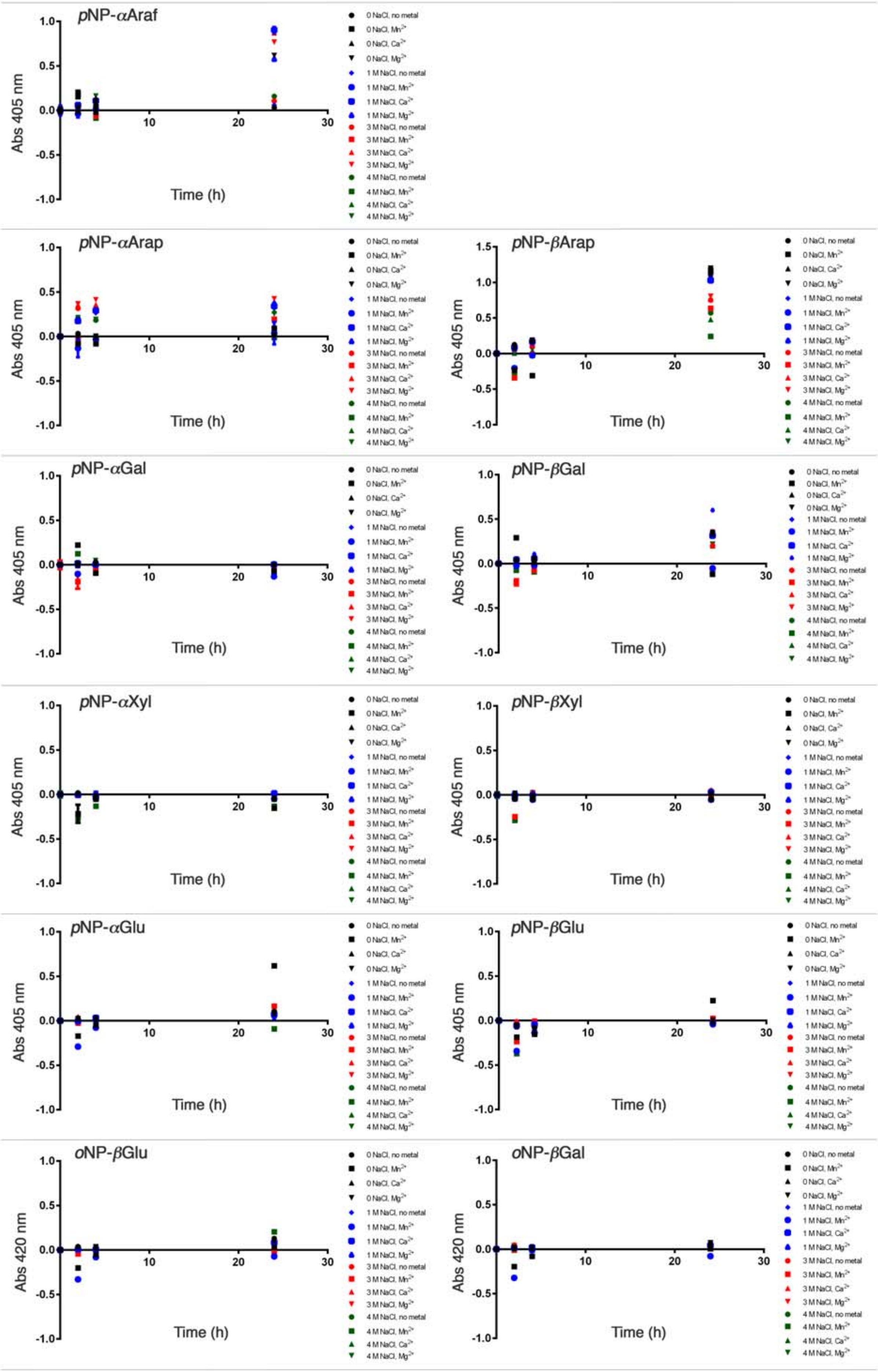
pNP-αAraf
pNP-αArap
pNP-βArap
pNP-αGal
pNP-βGal
pNP-αXyl
pNP-βXyl
pNP-αGlu
pNP-βGlu
oNP-βGlu
oNP-βGal
Abs 405 nm
Abs 420 nm
Time (h)
0 NaCl, no metal
0 NaCl, Mn2+
0 NaCl, Ca2+
0 NaCl, Mg2+
1 M NaCl, no metal
1 M NaCl, Mn2+
1 M NaCl, Ca2+
1 M NaCl, Mg2+
3 M NaCl, no metal
3 M NaCl, Mn2+
3 M NaCl, Ca2+
3 M NaCl, Mg2+
4 M NaCl, no metal
4 M NaCl, Mn2+
4 M NaCl, Ca2+
4 M NaCl, Mg2+

Supplementary Figure S2. Activity screen using chromophoric substrates. The inital screen was performed using eleven *p*- and *o*-nitrophenyl sugars, various NaCl concentrations (0, 1, 2 and 4 M NaCl), and different divalent cations (2 mM Mn^{2+}, Mg^{2+} or Ca^{2+}). The release of chromophore was measured as the absorbance at 405 nm for *p*-nitrophenyl and at 420 nm for *o*-nitrophenyl.

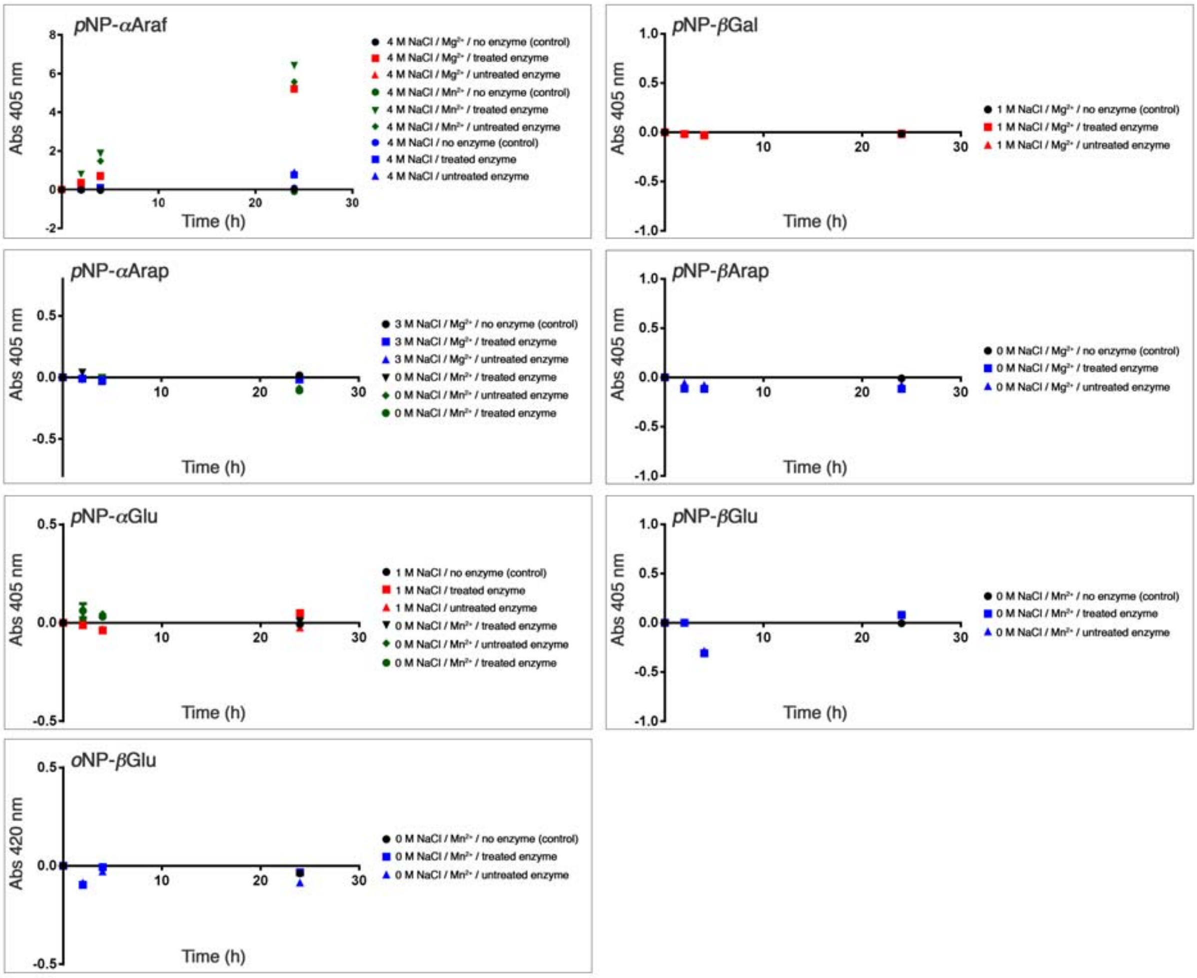

Supplementary Figure S3. Activity assay using a subset of substrates and conditions. Assays were performed with both EDTA/EGTA-treated and untreated protein.

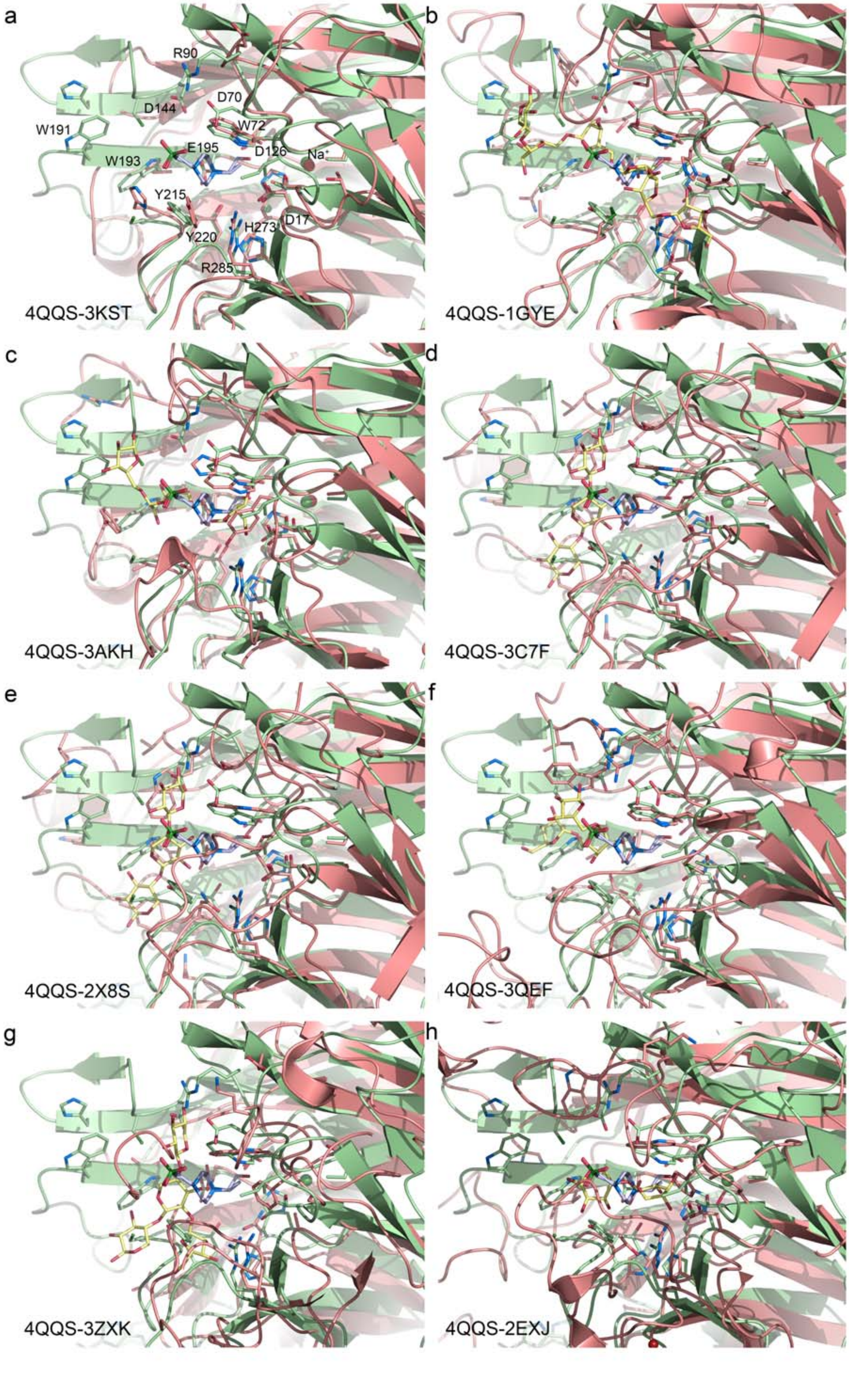
a
R90
D70
D144
W72
W191
E195
D126
Na+
W193
Y215
H273
D17
Y220
R285
4QQS-3KST
b
4QQS-1GYE
c
4QQS-3AKH
d
4QQS-3C7F
e
4QQS-2X8S
f
4QQS-3QEF
g
4QQS-3ZXK
h
4QQS-2EXJ

Supplementary Figure S4. Comparison of *Ho*Araf43 (green ribbon) with representative GH43 enzyme-carbohydrate complexes (red ribbon): (*a*) *B. thetaiotaomicron* endo-1,4-β-xylanase (3KST; r.m.s.d. 1.22 Å for 257 Cα atoms); (*b*) exo-α-1,5-L-arabinanase *Cj*Arb43A in complex with arabinohexaose (1GYE; Nurizzo *et al.*, 2002; r.m.s.d. 1.63 Å for 230 Cα atoms); (*c*) exo-α-1,5-L-arabinofuranosidase *Sa*Araf43A in complex with α-L-arabinofuranotriose (3AKH; Fujimoto *et al.*, 2010; r.m.s.d. 1.62 Å for 251 Cα atoms); (*d*) *B. subtilis* arabinoxylan α-1,3-L-arabinofuranohydrolase in complex with xylotriose (3C7F; Vandermarliere *et al.*, 2009; r.m.s.d. 1.88 Å for 229 Cα atoms); (*e*) *B. subtilis* endo-α-1,5-L-arabinanase Abn2 in complex with arabinotriose (2X8S; de Sanctis *et al.*, 2010; r.m.s.d. 1.71 Å for 219 Cα atoms); (*f*) *C. japnicus* α-1,2-L-arabinofuranosidase Abf43A in complex with α-1,3-arabinofuranose-substituted arabinofuranoside-substituted α-1,5-L-arabinotriose (3QEF; Cartmell *et al.*, 2011; r.m.s.d. 1.70 Å for 231 Cα atoms); (*g*) *H. insolens HiAXHd3* in complex with xylotriose (3ZXK; McKee *et al.*, 2012; r.m.s.d. 2.18 Å for 242 Cα atoms); (*h*) *G. stearothermophilus* β-xylosidase XynB3 in complex with xylobiose (2EXJ; Brüx *et al.*, 2006; r.m.s.d. 1.74 Å for 268 Cα atoms). Selected amino acids in *Ho*Araf43 have been added to panel (*a*).
